# The American Dental Association

**Published:** 1881-08

**Authors:** 


### THE AMERICAN DENTAL ASSOCIATION.

Another meeting of this grand old body has come and gone, and notwithstanding the fact that the session just passed was convened almost one month earlier than the constitutional date the attendance was large, and the labor performed brought forth fruits quite as excellent as any of its predecessors. There were fears in the minds of many of its members that this irregularity in the time of meeting would prove detrimental, if not fatal, to the interests of the association. It is gratifying to know that the souls of those who gather around the proud standard of our profession are made of stuff too stern to be turned away from their love by a fickle breeze.

The American Dental Association has ridden through many a storm and narrowly missed the wrecking rocks of contention, and it is safe to say that to-day her power and influence are greater than ever before. One of the best things accomplished at this last meeting was the selection of an Ohio man, Dr. H. A. Smith, of this city, for its President, and another in selecting Cincinnati, O., for its next place of meeting. Welcome!

